# The Therapeutics of Tea and Coffee

**Published:** 1882-07-20

**Authors:** F. E. Stewart

**Affiliations:** N. Y. City


### THE THERAPEUTICS OF TEA AND COFFEE.

BY F. E. STEWART, M. D., PH. G., N. Y. CITY.

Tea and coffee are used as beverages throughout the civilized world, and their effects have been studied with no little interest. The fact of their universal use has been employed as an argument to prove that man has a special need of them. · But this can hardly follow, for a perfect development can be reached without their aid. That they affect the system, there can be no manner of doubt, but whether this effect is beneficial or otherwise has formed a subject of much animated controversy.

To prove the value of a therapeutical agent it is necessary to first observe the results of experience in its use. Guided by that experience, experiments may now be advantageously undertaken to prove the scientific reasons for the results attained, and to prove or disprove the correctness of the observations which have been made empirically. The empirical and physiological researches with regard to the effects of tea and coffee have given them a comparatively certain and definite place as therapeutic agents. But more than empiricism and physiology was necessary to account for all of the actions attributed to them. Chemistry, however, has solved other problems pertaining to the study of the properties of these agents, and some of the reasons why they act so differently at different times on the same person. What, then, does empiricism, physiology and chemistry teach us about the therapeutic value of tea and coffee?

The experience of those who use coffee and tea show us that they are stimulating in their action, and that coffee is more so than tea. Both are asserted by some to be nutritious, which may, or may not be attributed to the fact that they remove the sense of fatigue and hunger, and allay the mental unrest produced by exhaustion and anxiety.

If used to excess they derange the organs of digestion and cause acidity, eructations, flatulence, pyrosis, etc. Functional disturbances of the nervous system are also caused by their excessive employment, and headache, vertigo, tinnitus aurium, and confusion of mind are the results.

A cup of coffee after dinner facilitates digestion. Those who take a cup of coffee in the morning suffer with a headache if they neglect it. Coffee is a laxative, tea is an astringent. The use of either frequently produces wakefulness.

The above is what is taught empirically with regard to the effects of tea and coffee. But the reason why these effects are produced must be looked for beneath the surface of empiricism, and now science has before her the important question to decide. Chemistry and physiology are appealed to for the solutions. Will the chemical composition and physiological actions of tea and coffee account for the results of experience in their use?

Tea and coffee are stimulants. Chemistry tells us they both contain volatile oils to which their aroma is due; physiology teaches us the functions of the nervous system, and we know that volatile oils stimulate the nerves. Both tea and coffee are asserted to be nutritious. Physiology has revealed that the products of waste are elminated from the system by the organs of excretion, and that much of this waste finds its way out through the urinary apparatus in the form of urea. Some agents are known to decrease the amount of urea eliminated. These agents are classed with the nutrients for that reason, though they do not necessarily take into the blood with their administration any considerable amount of nutritious material. It is said that tea and coffee decrease the amount of urea, and that, therefore, they belong to the nutrients. They allay mental unrest and remove the sense of fatigue. In doing so they but share with the other stimulants a property common to a greater or less extent to them all. Alcohol will do the same thing. Stimulants, as a rule, if taken in small doses, promote the appetite and increase the digestive power by stimulating the gastric follicles.

But if used to excess tea and coffee derange the organs of digestion and excite functional disturbance of the nervous system. This is also accounted for both chemically and physiologically. Both contain tannin. The mucus of the stomach plays the part of a ferment and acts on the starch taken as a constituent of the food, and it undergoes the acetic fermentation. This process is greatly facilitated by the presence of a quantity of weak alkaline solution. The eructations are gases formed by the chemical decomposition. The headache, vertigo, confusion of mind, etc., are the effects produced by over-stimulation of the nervous system.

A cup of coffee after a meal facilitates digestion, and those who take it in the morning suffer with headache for their neglect if they omit their ordinary cup. The effects of stimulants on the digestive functions have already been referred to. The stimulant also hastens the intestinal movements, and assists the morning evacuation, which, if retained, causes headache from the reflex irritation on the brain, due to the presence of fecal matter in the rectum.

Coffee is a laxative, but tea is an astringent. How can this be accounted for? It has been observed that the effects of tea and coffee are thus far identical, but in this they differ. As usually prepared, tea is subjected to long boiling, which extracts a large amount of the tannin from the leaves. Coffee, on the contrary, is usually prepared by infusion, and is either not boiled at all, or is subjected to the action of water but a short time before used. The amount of the tannic acid present accounts for the difference of effect. There are other reasons, but this one is sufficient for the illustration.

The similar effects of these two beverages have also been answered by chemistry. Tea and coffee derive their activity not only from the volatile oil which they contain, but from a very important constituent known by the names of caffeine, or theine. The effect of this alkaloid on the economy when isolated, is very similar to that of tea and coffee. Relative to its physiological action, Bartholow reports as follows:

"Caffeine, in small medicinal doses, promotes appetite, increases the digestive power by stimulating the gastric glands, and relaxes the bowels slightly. On the heart it exerts at first a decided stimulant action, and raises the arterial tension; but these effects are succeeded by weakened cardiac movements and diminished blood-pressure, cardiac muscle, and its contained ganglia being both probably paralyzed by it. Respiration ceases before the heart stops in animals poisoned by caffeine."

"As regards the cerebral effects, it may be stated that, at first, drowsiness occurs; but this is soon followed by wakefulness, excitement, muscular trembling, confusion of mind, hallucination, and delirium. The cerebral effects terminate in deep sopor, but this is probably the result of exhaustion. Rise of temperature, convulsions, general paralysis, occur when toxic doses are administered to animals; but the temperature declines when paralysis supervenes." supervenes."

The point of this article is to call attention to the importance of studying therapeutics as directed toward the drug. Every drug has activities pecular to it, and therapeutics can never be a science until the reasons for those activities can be accounted for. This can only be accomplished by first studying the drug empirically, then physiologically, and chemically. Physiological therapeutics are dependent on the knowledge obtained from empiricism to guide its researches. and chemistry is essential in the determination of the active principles of drugs — *Therapeutic Gazette.*

